# Preoperative predictive factors of carcinoma in situ in the normal‐appearing mucosa in patients who underwent an initial transurethral resection for non‐muscle‐invasive bladder cancer under white light cystoscopy

**DOI:** 10.1002/cnr2.1321

**Published:** 2020-11-11

**Authors:** Koji Iinuma, Kazuya Yuhara, Hiroto Kotaka, Kaori Ozawa, Daiki Kato, Manabu Takai, Keita Nakane, Kosuke Mizutani, Tomohiro Tsuchiya, Takuya Koie

**Affiliations:** ^1^ Department of Urology Gifu University Graduate, School of Medicine Gifu Japan; ^2^ Department of Urology Japanese Red Cross Takayama Hospital Takayama Japan

**Keywords:** carcinoma in situ, non‐muscle‐invasive bladder cancer, random biopsy, white light cystoscopy

## Abstract

**Background:**

Although few studies evaluated the significance of random biopsies under white light cystoscopy (WLC) in patients with non‐muscle‐invasive bladder cancer (NMIBC), the findings are controversial.

**Aim:**

This aim of this study was to evaluate what kind of preoperative covariates were useful as predictive factors in detecting carcinoma in situ (CIS) from normal‐appearing mucosa using random bladder biopsies under WLC.

**Methods and Results:**

A total of 229 patients with NMIBC underwent initial TUR followed by random biopsies under WLC at Red Cross Takayama Hospital between 2007 and 2016. These patients underwent TUR with complete resection of intravesical visible tumors followed by random biopsies of normal‐appearing mucosa. In this study, random bladder biopsies of normal‐appearing urothelial mucosa, excluding abnormal mucosa, were carried out with a cold punch in the selected intravesical sites. The covariates included age, gender, the urine cytology result, presence of an abnormal mucosa, number of tumors, size of the largest tumors, configuration of the tumor, and tumor type. Abnormal mucosa was defined as reddish or mossy areas at the time of TUR under WLC. The primary endpoint was to determine what kind of preoperative covariates were useful as predictive factors in detecting CIS from normal‐appearing mucosa using random bladder biopsies under WLC. Finally, 212 patients were evaluated, and 67 patients (31.6%) were diagnosed with CIS from normal‐appearing mucosa. In univariate analysis, positive urine cytology, abnormal mucosa, and the number of tumors were significantly associated with concomitant CIS. On multivariate analysis, positive urine cytology and abnormal mucosa were significantly associated with CIS.

**Conclusion:**

The patients who were diagnosed with positive urine cytology or abnormal mucosa by WLC are ideal candidates for TUR followed by random biopsy of normal‐appearing mucosa.

## 1 INTRODUCTION

Bladder cancer is the 11th most commonly diagnosed cancer worldwide.^1^ Of these, non‐muscle‐invasive bladder cancer (NMIBC) accounts for about 70% to 80% of all bladder cancers.^2^ Transurethral resection (TUR) is the initial step in treating NMIBC. The aim of TUR is to establish the histologic diagnosis, determine the tumor stage and grade, and remove all visible tumors including complete resection of all visible papillary tumors with concomitant biopsy of suspicious flat lesions.^3^ However, complete TUR is difficult to accomplish in a significant number of cases, especially under white light cystoscopy (WLC), because the detection rate with CIS under WLC is between 58% and 79%^3^, ^4^ Hara et al reported that 7.2% of the patients with a negative urine cytology result had concomitant CIS in the normal‐looking mucosa.^5^ In addition, CIS lesions are usually macroscopically indistinguishable from the non‐cancerous mucosa and can exist far away from the visible tumors.^6^ Therefore, approximately 40% to 80% of Ta/T1 disease recurred after the initial treatment, and approximately 15% of NMIBC patients with high‐grade T1 NMIBC are likely to have high rates of recurrence and progression that leads to muscle invasion, metastasis, or death.^7^, ^8^ On the other hand, several reports have suggested that bladder biopsies may induce implantation of tumor cells at the biopsied mucosal site.^9^, ^10^ Therefore, the European Association of Urology (EAU) guidelines recommended that routine random biopsy should be taken from normal‐appearing mucosa in patients with positive urine cytology, high grade, or nonpapillary tumors.^11^ In addition, routine random bladder biopsies are not recommended if bladder mucosa has a normal aspect and negative urine cytology.^11^

Recently, photodynamic diagnosis (PDD) using a photosensitizer that includes hexaminolevulinate (HAL) or 5‐aminolevulinic acid (5‐ALA) or narrow‐band imaging (NBI) are useful techniques for the detection of malignant tumors and CIS.^3^, ^11^, ^12^ Indeed, these techniques have little toxicity, and the learning curve is short.^11^ Some studies showed that CIS and/or dysplasia are easily detected using PDD.^12^, ^13^, ^14^ According to the beneficial effect of PDD on the recurrence rate in patients with TUR, several randomized controlled trials (RCTs) demonstrated a decreased risk of bladder cancer recurrence in the short and long term.^15^ However, PDD had lower specificity than WLC,^10^ and there were no differences in progression and mortality rates compared with WLC.^15^ Conversely, an RCT demonstrated that when NBI was used during TUR, the overall results of this study were negative.^16^

Although few studies have evaluated the significance of random biopsies in patients with NMIBC under WLC, these findings are controversial.^5^, ^17^ Hence, we retrospectively evaluated which preoperative covariates were useful as predictive factors in detecting CIS from normal‐appearing mucosa using random bladder biopsies under WLC and identified the candidates who benefited from this procedure.

## 2 PATIENTS AND METHODS

### 2.1 Patients

A total of 229 patients with NMIBC underwent initial TUR followed by random biopsies under WLC at Red Cross Takayama Hospital between 2007 and 2016. Of these, 17 patients with newly diagnosed NMIBC for whom urine cytology examination was not performed preoperatively were excluded from this study. The enrolled patients underwent TUR with complete resection of visible bladder tumors, including reddish or mossy areas, followed by random biopsies of normal‐appearing mucosa. Resected tumors were pathologically diagnosed as stage Ta‐T1 NMIBC, according to the American Joint Committee on Cancer staging manual, seventh edition.^18^ The covariates included the patient age, gender, urine cytology, abnormal mucosa, the number of tumors, the size of the largest tumors, the configuration of the tumor, tumor type, pathological T stage, and tumor grade. Abnormal mucosa was defined as reddish or mossy areas at the time of TUR under WLC. If patients had multiple bladder tumors, the highest stage and grade were adopted for each patient's tumor stage and tumor grade at the time of TURBT and random biopsy under WLC.

This study was approved by the institutional review board of the Japanese Red Cross Takayama Hospital (IRB H29‐05).

### 2.2 Random biopsies

In this study, random bladder biopsies of normal‐appearing urothelial mucosa, excluding abnormal mucosa, were carried out with a cold punch in the selected intravesical sites including the anterior wall, posterior wall, right wall, left wall, dome, trigone, bladder neck, and/or prostatic urethra, after TUR under WLC. The abnormal mucosal appearance was diagnosed by each urologist.

### 2.3 Pathological evaluations

An experienced uropathologist at our institution performed the review of histopathological diagnosis for all patients. Voided urine samples were obtained from all patients before TUR and cytologically examined using the standard Papanicolaou staining. If the urine cytology was class III or greater, it was defined as positive in this study.

### 2.4 Follow‐up schedule

The enrolled patients were assessed by urine cytology and cystoscopy every 3 months for 2 years after the initial TUR, every 6 months for the next 3 years, and annually thereafter. Tumor recurrence was defined as the relapse of a bladder tumor with microscopic evidence of urothelial malignancy or the presence of malignant cells during urine cytology analysis. If the patients had a recurrence after TUR, we performed ultrasonography, computed tomography, and magnetic resonance imaging to diagnose disease progression, lymph node involvements, or distant metastasis.

### 2.5 Endpoints and statistical analysis

The primary endpoint was to determine the predictive factors in patients with NMIBC who are ideal candidates for additional random bladder biopsies of normal‐appearing mucosa. Statistical analysis was performed by the chi‐square test or Mann‐Whitney *U* test. Logistic regression analysis was used to determine the significance of the preoperative predictive factors of CIS. Data were analyzed using the Statistical Package for the Social Sciences software version 24.0 (IBM Corp., Armonk, New York). All tests were two‐sided, and *P* < .05 was considered statistically significant.

## 3 RESULTS

### 3.1 Patient characteristics

A total of 212 patients enrolled in this study. The pretreatment characteristics of the patients are shown in Table 1. Sixty‐seven patients (31.6%) were diagnosed with CIS from normal‐appearing mucosa, and 39 (18.4%) patients had concomitant positive urine cytology and CIS. Of the patients who were diagnosed with concomitant CIS from normal‐appearance mucosa, 25 (37.3%) and 42 (62.7%) had a solitary tumor and multiple tumors, respectively; 44 (65.7%) and 23 (34.3%) patients had low‐grade and high‐grade tumor, respectively; and 45 (67.2%) and 21 (31.3%) had pathological stage Ta and T1, respectively.

**TABLE 1:** Preoperative patients' characteristics

Covariate	
Age (year, median, interquartile range)	72.5 (65‐79)
Gender (number, %)	
Male	175 (82.5)
Female	37 (17.5)
Urine cytology (number, %)	
Positive	66 (31.1)
Negative	146 (68.9)
Abnormal mucosa (number, %)	
The patients who had normal‐appearing mucosa	161 (75.9)
The patients who had reddish or mussy areas	51 (24.1)
The number of tumors (number, %)	
Solitary	106 (50)
Multiple	106 (50)
The size of the largest tumors (number, %)	
<3 cm	183 (86.3)
≥3 cm	29 (13.7)
The configuration of the tumor (number, %)	
Papillary	193 (91)
Non‐papillary	19 (9)
The patients who diagnosed carcinoma‐in‐situ by random biopsy of the urinary bladder (number, %)	
Yes	67 (31.6)
No	145 (68.4)

The results of the univariate and multivariate analyses are listed in Table 2. In univariate analysis, positive urine cytology, abnormal mucosa, and the number of tumors were the significant predictors that were associated with concomitant CIS (Table 2). On multivariate analysis, positive urine cytology and abnormal mucosa were significantly associated with CIS.

**TABLE 2:** Univariate and multivariate analysis

	Univariate			Multivariate		
Covariates	OR	95% CI	*P* value	OR	95% CI	*P* value
Age	‐	‐	.73	‐	‐	‐
Gender						
Male	1 (ref.)	0.78‐3.35	.24	‐	‐	‐
Female	1.61					
Urine cytology						
Negative	1 (ref.)	3.21‐11.6	<.001	1 (ref.)	1.59‐7.54	.002
Positive	6.09			3.46		
The patients who had reddish or mussy areas						
No	1 (ref.)	11.0‐58.6	<.001	1 (ref.)	7.75‐51.1	<.001
Yes	25.4			19.9		
The number of tumors						
Solitary	1 (ref.)	1.17‐3.85	.018	1 (ref.)	0.52‐2.61	.71
Multiple	2.13			1.16		
The size of the largest tumors						
<3 cm	1 (ref.)	0.74‐3.67	.28	‐	‐	‐
≥3 cm	1.64					
The configuration of the tumor						
Papillary	1 (ref.)	0.63‐4.32	.31	‐	‐	‐
Non‐papillary	1.65					
Tumor type						
Pedunculated	1 (ref.)	1.23‐4.01	.010	1 (ref.)	0.45‐2.39	.94
Sessile	2.22			1.03		

Abbreviations: CI, confidence interval; OR, odds ratio.

## 4 DISCUSSION

Although the removal of all visible tumor tissue is crucial in the treatment of NMIBC, complete resection of multiple visible lesions, especially of CIS, was often difficult during TUR. Generally, CIS is often flat, high‐grade, and multifocal lesions.^6^ Therefore, CIS are usually macroscopically indistinguishable from normal‐appearing mucosa and can exist far from visible tumors.^6^ Random biopsies in patients with NIMBC is not recommended in the EAU and the National Comprehensive Cancer Network guidelines.^11^, ^19^ In the European Organization for Research and Treatment of Cancer protocol 30 863, random biopsies from normal‐appearing mucosa during TUR may not contribute to the staging or the choice of adjuvant therapy after transurethral resection because of the low incidence of CIS.^20^ Conversely, May et al reported that 12.4% of the patients with NMIBC had diagnosed CIS from the normal‐appearing mucosa.^9^ Therefore, the clinical significance of random biopsies from normal‐appearing mucosa remains controversial.^7^

Indeed, bladder biopsy carries the risk of bleeding, infection, and possible implantation of tumor cells at the biopsy mucosa.^9^, ^10^ However, approximately one‐sixth of patients with Ta or T1 tumors appeared to have concomitant CIS that was detectable by random bladder biopsies. In this study, 31.6% of the patients were diagnosed with CIS from the normal‐appearing mucosa. This changed the postoperative treatment and follow‐up schedule in such patients.^17^ Furthermore, early diagnosis of CIS by random biopsies and optimal postoperative therapy such as intravesical instillation of Bacillus Calmette‐Guérin (BCG), may significantly improve the prognosis of the patients with concomitant CIS.^17^ It was reported that TUR with subsequent BCG instillation was more effective in preventing the recurrence of TaT1 tumors than TUR alone or TUR combined with chemotherapy.^21^ A meta‐analysis demonstrated the benefits of adjuvant BCG on progression.^21^ Based on data from 24 trials involving 4863 patients, 9.8% of the patients receiving BCG showed tumor progression compared to 13.8% of controls, which represents a relative risk reduction of 27%. Therefore, any nonvisible residual tumors of the bladder after the initial TUR may be eradicated with adjuvant BCG treatment.^22^

Recently, it was reported that PDD after intravesical instillation of 5‐ALA or HAL was better in tumor detection than WLC.^23^ In a systematic review and meta‐analysis, PDD had higher sensitivity than WLC in the pooled estimates for analyses at both patient level (92% vs 71%) and biopsy level (93% vs 65%).^12^ A systematic review and analysis of 14 RCTs demonstrated a decreased risk of bladder cancer recurrence in the short and long term.^11^ A prospective randomized study demonstrated a delay in time to tumor recurrence after PDD‐assisted TUR (16.4 months) compared to WLC alone,^24^ and a meta‐analysis of 634 patients showed a recurrence rate of 34.5% with PDD at 12 months and 45.4% with WLC.^23^ On the other hand, PDD had lower specificity than WLC in the pooled estimates for patient and biopsy level analyses (72% vs 95% and 81% vs 95%, respectively).^12^ The overall incidence of T2‐T4 tumors was 6.1% in the patients who were examined by WLC and 3.1% in the patients who were diagnosed by PDD (*P* = .066) .^25^ There were no differences in tumor progression and mortality rates.^11^, ^12^, ^23^ Currently, the benefit of using PDD during TUR in reducing tumor recurrence and progression in the long term remains unclear.^25^

In this study, positive urine cytology and abnormal mucosa were significantly associated with the diagnosis of CIS. Several studies reported that urine cytology has a high sensitivity for the diagnosis of high‐grade urothelial carcinoma such as CIS but a low sensitivity for low‐grade urothelial carcinoma.^6^, ^26^, ^27^, ^28^ Although the bladder CIS is characterized by reddish, coarse, mussy, and edematous areas under WLC, some of the lesions have a normal appearance, and these areas are not necessary for detecting CIS.^29^ Therefore, the presence of bladder CIS at not only tumors or abnormal mucosa but also normal‐appearing mucosa may be associated with positive urine cytology in this study.

Our study has several limitations. First, it is a retrospective study that was performed at a single institution, and a relatively small number of patients were enrolled in this study. Second, the evaluation of reddish or mossy areas of the mucosa by random biopsy under WLC may vary among surgeons. Finally, the biopsies with prostatic urethra were excluded from this study.

In conclusion, the present study showed that positive urine cytology and abnormal mucosa were significantly associated with the presence of CIS that was collected from normal‐appearing mucosa under WLC. The patients who were diagnosed with positive urine cytology or abnormal mucosa by WLC are ideal candidates for TUR followed by random biopsy of normal‐appearing mucosa.

## CONFLICT OF INTEREST

The authors have stated explicitly that there are no conflicts of interest in connection with this article.

## AUTHOR CONTRIBUTIONS

All authors had full access to the data in the study and take responsibility for the integrity of the data and the accuracy of the data analysis. *Conceptualization*, K.I., T.K.; *Methodology*, K.I., T.K.; *Investigation*, K.Y., H.K., K.O.; *Formal Analysis*, K.I., K.M.; *Resources*, D.K., M.T., K.N.; *Writing ‐ Original Draft*, K.I; *Writing ‐ Review and Editing*, T.K., K.M.; *Supervision*, T.T.; *Project Administration*, T.K.; *Funding Acquisition*, K.M.

## INFORMED CONSENT

For this type of study formal consent is not required. Pursuant to the provisions of the ethics committee and the ethic guideline in Japan, written consent was not required in exchange for public disclosure of study information in the case of retrospective and/or observational study using a material such as the existing documentation.

## ETHICS STATEMENT

All procedures performed in studies involving human participants were in accordance with the ethical standards of the institutional research committee and with the 1964 Helsinki declaration and its later amendments or comparable ethical standards.

## Data Availability

The data that support the findings of this study are available on request from the corresponding author. The data are not publicly available because of privacy or ethical restrictions.
